# “It’s all in the moment”: a mixed-methods study of elementary science teacher adaptiveness following professional development on knowledge generation approaches

**DOI:** 10.1186/s43031-022-00052-3

**Published:** 2022-04-05

**Authors:** Catherine Lammert, Brian Hand, Jee Kyung Suh, Gavin Fulmer

**Affiliations:** 1 Department of Teacher Education, Texas Tech University, 3002 18th St, #351, Lubbock, TX 79409 USA; 2 Department of Teaching and Learning, University of Iowa, N297 Lindquist Center, Iowa City, IA 52242 USA; 3 Department of Curriculum and Instruction, University of Alabama, BOX 870232, AL 35401 Tuscaloosa, USA

**Keywords:** Elementary science education, Professional development, Instructional planning

## Abstract

This special edition is based on the revelation that “the lessons learned and unlearned during COVID-19 grant us an unparalleled opportunity to reflect.” Here, we reflect on lessons learned related to teacher adaptiveness. We examined how the COVID-19 pandemic demonstrated the adaptiveness necessary for teachers to knowledge generation approaches aligned with the Next Generation Science Standards. First, we outline a three-year professional development program focused on knowledge generation approaches. We present findings from teachers’ experiences teaching science from 2019 to 2021, collected through consecutive form explanatory mixed-methods analysis involving written responses to vignettes (*n* = 474) and classroom observations (*n* = 58). Then, using an individual teacher case study, we explore how the shift to virtual teaching was supported by adaptiveness. Results suggest a significant relationship between teacher adaptiveness and the use of knowledge generation approaches. We conclude with implications for elementary science teacher professional development and present questions for further research on adaptiveness.

**Supplementary Information:**

The online version contains supplementary material available at 10.1186/s43031-022-00052-3.

## Introduction

The COVID-19 pandemic led to many changes in science education. One notable shift was the movement to remote, virtual, and hybrid learning in the elementary grades during the 2019–2020 and 2020–2021 school years (Engelbrecht et al., 2020). This shift was enabled by teachers’ use of instructional technologies that they were not always prepared to use (Hodges et al., 2020). Thus, this situation created a unique opportunity to ask questions about elementary science teachers’ ability to adapt as they provided virtual learning opportunities and altered well-worn routines (Bingimlas, 2009).

In particular, science teaching during the pandemic has required *adaptiveness*, defined as the ability to support student thinking through the use of language tools inside the context of scientific inquiry (Allen et al., 2013). The notion that effective teaching requires adaptiveness was magnified by COVID-19, but it had already emerged prior to the pandemic (Vaughn & Parsons, 2013). The Next Generation Science Standards (NGSS Lead States, 2013) and statements from high-impact organizations (e.g., Organization for Economic Cooperation and Development (OECD), 2018) promote knowledge generation approaches to teaching that require adaptiveness. In knowledge generation approaches, students ask questions, plan and conduct investigations, and evaluate ideas with one another (Hand et al., 2021; McNeill et al., 2016; Tang, 2020). Knowledge generation approaches rely on tools such as language, negotiation, and dialogue to teach science, and the use of these tools requires teacher adaptiveness (Collie et al., 2020; Loughland & Alonzo, 2018; Parsons et al., 2018). During the COVID-19 pandemic, the demand for teachers to be adaptive increased further, as they grappled with the well-documented challenges of effectively integrating technology in their teaching (Bingimlas, 2009) so that they could use knowledge generation approaches.

While we acknowledge the challenges of teaching in the context of a pandemic, we also see possibilities that these unique circumstances uncovered. This consecutive explanatory mixed-methods study (Creswell, 2013) is situated within a three-year study of 119 elementary teachers’ science teaching across two U.S. states, with an emphasis on knowledge generation approaches for science teaching. The study began in spring 2019, before the pandemic, and continued through summer 2021, allowing us to trace shifts in teachers’ ideas and orientations over time. This paper’s purpose is to investigate how science teachers engaged in adaptiveness as they used knowledge generation approaches during the COVID-19 pandemic. In doing so, we identify both difficulties and affordances as we explore the teacher adaptiveness needed to eact NGSS-aligned (2013) teaching in remote, virtual, and hybrid settings.

## Background

First, we review what is known about elementary science teachers’ ability to provide remote, virtual, and hybrid teaching experiences. Then, we review knowledge generation approaches for teaching, and we suggest how they might be supported by teachers’ adaptive use of technology. We conclude by reviewing theories of adaptiveness to demonstrate how adaptiveness might be the link between teachers’ ability to use technologies inside knowledge generation approaches, particularly in the remote, virtual, and hybrid learning formats that the COVID-19 pandemic necessitated.

### Elementary science teachers’ knowledge and use of instructional technology

In the U.S., most elementary (i.e., early childhood through fifth grade/ages 4–11) students attended school face-to-face in January of 2020, when the first COVID-19 cases were reported (Martin et al., 2020). In these settings, teachers used instructional technologies to enhance in-person learning in classrooms where children could physically work together. Technology created additional possibilities for interaction, access of information, and writing/ composition. In their review of literature, Bingimlas (2009) reported that despite occasional challenges and questions about how to ensure that technology supports content learning (e.g., van Broekhuizen, 2016), this type of technology integration was largely successful.

The pandemic required a sudden shift to synchronous and asynchronous remote learning. In these virtual settings, teachers had a fundamentally different purpose for using instructional technology: Now, it was necessary to communicate with students, facilitate student-to-student communication, and manage learning resources, texts, and assignments (Svrcek et al., 2021). Most elementary teachers did not have the time or advanced preparation that instructors of online courses typically receive before teaching virtually (Gewin, 2020; Hodges et al., 2020). In fact, it may be unfair to judge the potential of online elementary science learning based on the teaching conducted during the early months of the COVID-19 pandemic, which was characterized by a rushed shift in formats (Hodges et al., 2020). Similarly, it is unclear whether elementary science teachers were able to create effective learning environments or use knowledge generation approaches in remote, virtual, and hybrid teaching.

### Knowledge generation approaches

Knowledge generation approaches are rooted the view of students as active sense-makers (Hand et al., 2021; Organization for Economic Cooperation and Development (OECD), 2018). Teachers can support knowledge generation by thinking aloud alongside students during investigations to model the process of evaluating the quality of evidence for a claim (Boon & Van Baalen, 2019; Tang, 2020). In science education, tools such as language, negotiation, and dialogue are particularly valuable in supporting knowledge generation. In classrooms where students are physically present together, discussing, writing, and sharing ideas is relatively intuitive. However, in remote, virtual, and hybrid learning, these interactions must be facilitated by technology with teacher support. Knowledge generation approaches are thought to require more sophisticated planning practices (Contreras et al., 2020) to allow teachers to consider what their students already know and gather resources to increase their understanding (McNeill et al., 2016). Notably, the utility of language tools is made apparent in the moment as students share their reasoning and teachers decide how to respond, which suggests a need for adaptiveness.

### Teacher adaptiveness

**Fig. 1:**
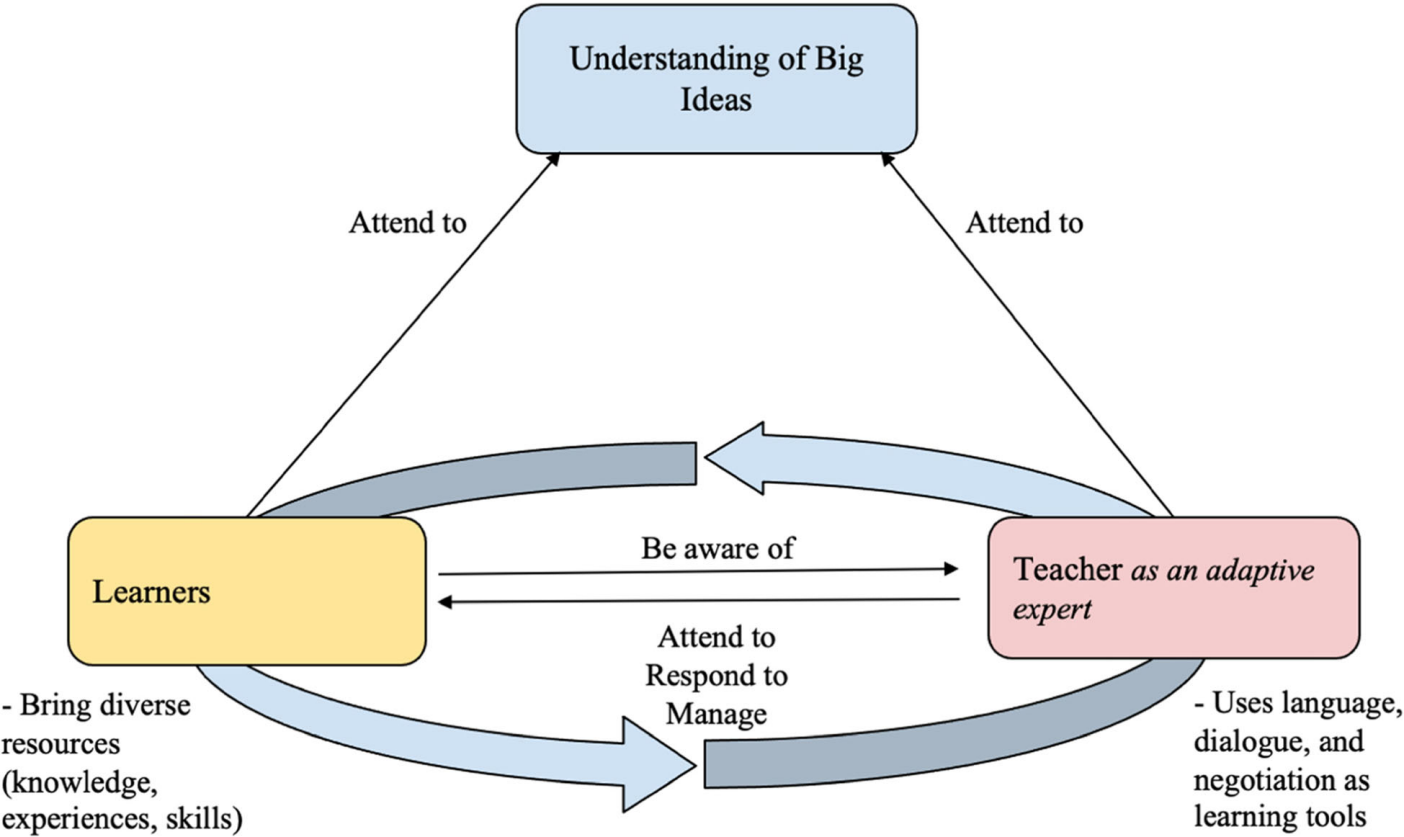
Model of Elementary Science Teacher Adaptiveness

For many, the COVID-19 pandemic threw teachers and students into an unplanned situation requiring adaptiveness, which is the ability to support student thinking within scientific inquiry through tools such as language, dialogue, and negotiation (Allen et al., 2013; Loughland & Alonzo, 2018). However, a review by Parsons et al. (2018) suggests that since the 1970s, researchers have argued that adaptiveness is a foundational element of effective teaching. The 2015 Program for International Student Assessment (PISA) data indicated that across eight nations and thousands of schools, science teacher adaptiveness significantly was significantly positively associated with teacher self-efficacy, student self-efficacy, and student science achievement (Collie et al., 2020). Professional, pedagogical, and content knowledge all remain important, but emerging evidence indicates that NGSS-aligned (2013) science teaching requires adaptiveness (Allen et al., 2013; Collie et al., 2020). The model of elementary science teacher adaptiveness that drove this research is presented in Fig. 1. This model is based on the work of Hatano and Inagaki (1986), who proposed a framework for understanding teachers’ decision making that contrasts routine expertise with adaptive expertise. Routine experts are knowledgeable of how to apply prepared responses to challenges, but their work is less innovative. Their teaching typically corresponds to traditional learning environments dedicated to rote memorization and skills mastery. In contrast, adaptive experts embed options in their instructional plans with the intention of supporting students’ ideas and questions. In applying Hatano and Inagaki’s (1986) framework to the context of elementary science teaching, we have developed the model (Fig. 1) to show how adaptive expertise can be operationalized in professional development and research.

In this model, the teacher’s role as adaptive expert is to move fluidly between learners and the big ideas of science. This conceptualization of adaptiveness is particularly relevant to knowledge generation approaches (Hand et al., 2021), where teachers must make moment-to-moment decisions about what questions to ask and how to respond to students’ ideas. The current study explored the possibility that adaptiveness is particularly important in uncertain teaching contexts, such as the remote, virtual, and hybrid formats constructed during the COVID-19 pandemic, in which instructional technology was necessary for knowledge generation approaches to teaching science.

## Research question

In teaching altered by the COVID-19 pandemic, how does elementary science teacher adaptiveness relate to the use of knowledge generation approaches?

## Methods

This report is situated within a three-year study of 119 elementary teachers’ science teaching. This research focuses specifically on the period from January through June 2020, during the beginning of the COVID-19 pandemic’s impact, through May 2021, during the second school year of pandemic-altered teaching. During this time frame, 119 teachers participated in professional development on knowledge generation approaches.

### Sampling procedures

Elementary (Kindergarten through 5th grade) science teachers were recruited for the professional development across two U.S. states. In these states, representatives of the local education agencies contacted districts, schools, and individual teachers about the professional development program. Both states had recently adopted the NGSS (2013). Twenty-nine teachers were recruited from a state in the U.S. Southeast, and 80 teachers were recruited from a state in the U.S. Midwest. Three were men and 116 were women. Eight taught kindergarten, 13 taught 1st grade, 13 taught 2nd grade, 33 taught 3rd grade, 36 taught 4th grade, 16 taught 5th grade. The mean number of years of teaching experience was 13.9. All teachers provided written informed consent to participate in the research in accordance with university Institutional Review Board procedures. All names are abbreviated pseudonyms.

### Professional development context

During summer 2019, all 119 teachers engaged in 6 days of in-person professional development based on the Science Writing Heuristic (SWH; Keys et al., 1999).

*Days 1–2:* The first 2 days defined the fundamental elements of knowledge generation approaches. Teachers defined “language,” “dialogue” and “negotiation” in small groups, then negotiated their definitions with other groups. Consistent with our knowledge generation approach framework (Keys et al., 1999), language was defined as including speech, writing, reading, and listening, and involving multiple modes of representation (e.g., pictures, graphs). Dialogue was defined as purposeful interaction with others to clarify science concepts and terms. Negotiation was defined as the process of supporting claims with evidence, comparing claims to others’, and evaluating the strength of different arguments.

*Days 3–4:* The second 2 days were immersive and allowed teachers to experience pedagogies aligned with knowledge generation approaches from students’ perspective. They also included extended discussion of adaptiveness, which we defined for teachers as the ability to flexibly question and respond to students ideas during science investigations and inquiry by relying on language, dialogue, and negotiation (Hand et al., 2021).

*Days 5–6:* The third 2 days focused on preparation of NGSS-aligned units of study designed based on a knowledge generation approach. All 119 teachers then were supported by professional development consultants through single-session trainings and classroom observation during the subsequent school year. When instruction shifted from in-person to virtual in spring 2020, the professional development consultants continued supporting them through virtual visits and email consultations, depending on individual teachers’ needs.

In summer 2020, all participating teachers engaged in four-and-a-half days of virtual professional development. The content of the second summer’s professional development was designed to deepen teachers’ understanding of learning theory and adaptiveness, as well as their knowledge of the digital tools needed to use knowledge generation approaches opportunities in remote, virtual, and hybrid environments (Bingimlas, 2009). Again, the professional development consultants provided individualized support to all 119 teachers during the 2020–2021 school year, depending on the COVID-19 regulations governing each school and/or district related to in-person visitors. Some teachers were coached in person all year, others only were able to be coached in person in late spring 2021, and some were coached only virtually.

### Data sources

There were three sets of data sources: (A) four vignette tasks administered to all 119 participants, with two incomplete responses totaling *n* = 474; (B) implementation scores collected through observation of teachers (*n* = 58); and (C) an individual teacher case study drawn from interviews, observations, course artifacts, lesson plans, and email exchanges.

### Vignette tasks

During the virtual workshops conducted in June 2020, the whole group of participants (*n* = 119) responded to four vignette tasks. Vignettes are commonly used in the fields of education, business, and medicine to examine professionals’ judgement-making (e.g., Briggs, 2010). Here, the vignettes enabled us to analyze whether teachers who were able to respond with a more adaptive approach on the written vignette task also were able to implement more NGSS-aligned teaching, which would suggest a relationship between the two. This task involved written responses to a series of four scenarios. Respondents replied to these scenarios in writing (Supplementary Table 2). The vignettes were administered at a rate of one daily during the virtual summer 2020 workshop, before workshop content was provided that addressed the topics covered in the vignettes. The vignettes were based on a previously validated vignette instrument that focused on adaptiveness (Vogt & Rogalla, 2009) but were modified to fit the elementary teaching context (e.g., the focus was broadened to include all science topics, not just natural science) and the emphasis on language, negotiation, and dialogue as specific tools for science teaching.

### Implementation scores

To understand as much as we could about the limitations and possibilities afforded by the pandemic teaching context, an implementation score was sought through in-person and/or virtual observation of every participant in the January–June 2020 period. The implementation score involved the professional development consultants rating teachers from 1 to 3 on their use of seven NGSS-aligned teaching practices based on a scheduled observation of one science lesson. The seven practices in the implementation guide were determined to be consistent with knowledge generation approaches based on prior research (Hand et al., 2021) (see Supplementary Table 1 for the full implementation scoring guide). Three professional development consultants were trained on the use of the implementation rubric using classroom videos that they viewed, scored individually, and discussed with the research team. The professional development consultants were instructed to always observe the science lesson in its entirety before scoring or engaging with the teacher. Observed lessons ranged from 25 min to 55 min in length, with longer lessons typically occurring in upper elementary settings (3rd- 5th grade) and shorter lessons in early elementary settings (Kindergarten- 2nd grade). All 119 teachers were invited to participate in an observation, however, at this stage, many schools were new to virtual learning. Some teachers had concerns about sharing videos of online instruction with the research team, and many teachers were not familiar with the technology needed to do so. Ultimately, about half (*n* = 58) of the 119 teachers were able to be observed.

### Case study

A deeper look into the realities of teaching during a pandemic was needed, so a case study (Yin, 2013) model was chosen. In case study research, participant selection often reflects researchers’ goals, which can limit the utility of the research if these goals are not explained (Creswell, 2013). In this situation, our goal was to understand the contextual factors that could contribute quantitative trends that were emerging through our analysis of the vignettes and implementation scores. We also had to consider the ethics of asking teachers to participate in additional data collection during a teaching period that already was disrupted. Thus, we did not seek representativeness, but rather chose to identify a telling case (Mitchell, 1984) of a teacher who might expand our theorization of adaptiveness.

This teacher, who was selected after the June 2020 workshops, had previously agreed to participate in an additional case study, but we found that conducting research with beneficence during the pandemic required an ongoing negotiation of her involvement as she managed caring for herself and her own family, and teaching her own students, with her engagement with the research team. Ultimately, this teacher participated in two half-hour semi-structured interviews via Zoom, which were conducted at the beginning and midpoint of the school year to solicit her views and experiences related to virtual, hybrid, and in-person teaching. She also shared her class blog with the researchers, so we could view her daily interactions with her students. Furthermore, we conducted eight virtual observations and one in-person observation of her practice. Prior to the pandemic, during the 2019–2020 school year, she had shared two daily lesson plans with researchers, which became a baseline data point for understanding her planning process. To understand how the pandemic influenced her planning, she agreed to share more of her plans with researchers during the 2020–2021 school year. From September 21, 2020, through May 7, 2021, she shared 23 daily lesson plans with the research team. She also agreed to forward her email communication with her professional development consultant to us so that we could see how he was supporting her. Along the way, the research team checked in with her regularly via email and Zoom, asking questions such as: “How is your involvement in this study impacting you? How are you doing mentally, physically, and emotionally right now?” The purpose of these questions was to determine the appropriateness of her ongoing involvement in the study. In all, despite the physical distance that the pandemic created, this combination of data sources afforded a rich window into her thinking and experiences.

### Analysis

This consecutive-form, explanatory, mixed-methods analysis (Creswell, 2013) used embedded design to allow a relationship to emerge between qualitative and quantitative data sources. First, each of the four vignettes was scored using a three-point rubric, yielding a total score of 12 possible points (Supplementary Table 3). It was necessary to adapt the scoring system created by Vogt and Rogalla (2009) because the original vignette instrument only focused on one area of science (i.e., natural science) and it was a single scenario rather than four scenarios focused on tools such as language, negotiation, and dialogue. Further, the original scoring system simply rated teachers’ responses as (A) containing one or more marker of adaptiveness, or (B) not indicating adaptiveness. Consistent with our model of adaptiveness (Fig. 1) and interest in knowledge generation approaches, we expanded upon the work of Vogt and Rogalla (2009) to extend the scoring system to attend to three elements separately: the teacher’s role, the learner’s role, and the role of big ideas. The relationship between Vogt and Rogalla’s (2009) scoring categories and the revised scoring categories is demonstrated in Supplementary Table 3. Two raters scored the vignettes independently. Interrater reliability was calculated for each coding category, and was 91.4% for teacher’s role, 98.3% for learner’s role, and 96.6% for big ideas. Interrater reliability is reported (Table 1).

**Table 1:** Interrater Reliability for Vignette Scoring Procedure

	Teacher Role	Learner Role	Big Ideas	Total
**Scorer 1 Average**	77.6%	58.6%	74.1%	2.10
**Scorer 2 Average**	86.2%	60.3%	70.7%	2.17
**Variance**	−8.6%	1.7%	3.4%	.08
**Cross-Scorer Average**	82%	59%	72%	2.14

Then, the vignette responses were thematically analyzed. In analyzing vignette responses, Finch (1999) has argued that researchers must give particular attention to content that participants introduce that was not provided in the scenario, especially if this content is also mentioned by other participants. Accordingly, a first round of coding (Saldaña, 2013) was used to determine broad trends. One researcher re-read all responses and made notes in the margin regarding each category from the scoring system: the teacher’s role, the learner’s role, and the role of big ideas. In addition, to support trustworthiness of the conclusions (Creswell, 2013), the author used an “other” category to open-code comments that were pertinent to the research question but did not fit the previously established categories. The coding process, including examples of first and second round codes, is represented in Table 2.

**Table 2:** Thematic Coding of Vignette Responses

First Round: Initial Codes	Examples of Initial Code Notes	Second Round: Open Codes Expanded	Examples of Open Codes
**Teacher’s Role**	“the teacher should figure out what students know and create group structures”	**Time/Planning**	“You have be on your toes, but also have a hold on the material being negotiated”
**Learner’s Role**	“the students need to separate ideas/ evidence from the people who have those ideas”	**Technology**	“Use technology in between some of their dialogue to help answer questions”
**Big Ideas**	“try it with basic ideas before digging in to science content”	**Developmental Ability/Grade Level**	“In first grade, the teacher needs to help guide”
**Other**	“it’s okay to take the time to do more negotiation”	**Alignment with Teacher in Scenario**	“I feel like Naomi! I am still figuring this out.”

Subsequent quantitative analysis focused on the 58 implementation scores, provided on a scale of 1–3, which were compared with those same teachers’ overall scores on the vignette task, which was scaled from 0 to 12 (three points possible per vignette; four vignettes). The direction and strength of the relationship were analyzed using descriptive statistics and ANOVA (Mertens, 2015). This analysis suggested the existence of a significant positive relationship between teacher adaptiveness and teachers’ ability to use knowledge generation approaches, which we aimed to understand through the case study analysis.

Consistent with consecutive form explanatory approaches (Creswell, 2013), the goal of the qualitative case study analysis was to use a situated example to examine this relationship’s dynamics in a real-world context. First, the lesson plans’ length was analyzed using descriptive quantitative methods, then case study data – including transcripts of interviews, written lesson plan commentary, and emails – were analyzed thematically using a constant comparative method (Strauss & Corbin, 1998), with two rounds of inductive, open coding. Consistent with the research question, the first round of the coding process focused on identifying evidence of adaptiveness and the construction of knowledge generation approaches, as well as gathering disconfirming evidence in areas where these were not present. During the second round of the coding process, this evidence was moved back into a chronological organization so that patterns across time and in relation to teaching format (remote, virtual, and hybrid) could be apparent.

## Results

The results are organized beginning with the analysis of the vignettes, including overall trends, qualitative thematic analysis, and quantitative analysis. Then, case study results are presented.

### Vignette analysis results: overall trends

Analysis of participants’ responses to the vignettes revealed several patterns. First, in response to the general prompt, teachers were most likely to state their own role (82%) in adaptive teaching, followed by the importance of engaging with big ideas (72%). They were least likely to discuss the learner’s role (59%). When a particular tool (e.g., language, dialogue, negotiation) was suggested, teachers continued to name their own role in classroom practice most often. However, when the vignette directed them toward particular tools, their attention to big ideas dropped dramatically (18% for language, 16% for negotiation, and 20% for dialogue, respectively). Attention to the learner’s role was highest (65%) when the language tool was the vignette’s focus. Table 3 shows the overall scores on the vignette tasks.

**Table 3:** Summary of Adaptiveness Vignette Scores: Number and Percent of Respondents Addressing Each Area (*n* = 474)

	Teacher Role		Learner Role		Big Ideas		Total		Average Score per Vignette
	N of resp.	Percent	N of resp.	Percent	N of resp.	Percent	N of resp.	Percent	
**Vignette 1: General** **(*n*** **= 119)**	98/119	82%	70/119	59%	86/119	72%	84/119	71%	2.14/3
**Vignette 2: Language** **(*n*** **= 119)**	102/119	86%	77/119	65%	21/119	18%	67/119	56%	1.67/3
**Vignette 3: Negotiation** **(*n*** **= 118)**	82/118	70%	66/118	56%	19/118	16%	55/118	47%	1.42/3
**Vignette 4: Dialogue** **(*n*** **= 118)**	99/118	84%	54/118	46%	24/118	20%	59/118	50%	1.49/3

Note. The scoring criteria is presented in Supplementary Table 3

### Vignette analysis results: qualitative

The qualitative results are presented thematically around each a priori code: the teacher’s role, the learner’s role, and big ideas. Then, two additional second-round themes are described.

#### Teacher role

The role of the teacher was described most often of the three coding categories. The teacher’s role was mentioned in 82% of the responses to Vignette 1: General, 86% of responses to Vignette 2: Language, 70% of responses to Vignette 3: Negotiation, and 84% of responses to Vignette 4: Dialogue. Across all four vignettes, respondents wrote in ways that described the challenges of adaptive teaching. Responding to Vignette 3, LS wrote “Negotiation is a hard one to learn. Sometimes I struggle with thinking quick enough to keep the negotiating going. It definitely is something you have be on your toes for.” Respondents mentioned that being familiar with the science content and preparing questions in advance can support their ability to adapt instruction, but it is impossible to predict everything that might happen. Some teachers reported comfort with this. As a prime example, TB wrote,

I am more of an ‘off the cuff’ teacher. I feel how my students are when they come in the room to see where the day is going. When we do [investigations], then I encourage more talking and coming up with a plan on how to create it. But even when we do research, I leave it up to them to see where they want to go in their research as long as they cover the big idea (Vignette 4).

Other teachers acknowledged similar challenges with adaptiveness and preparation, but some expressed that they were less comfortable with the process. For example, KB wrote, “When it comes to negotiations you have to let the kids take the lead and let the conversation go where it may” but elaborated, “I would also tell Emma I’m with her on this, I need to make better use of my negotiations. I feel it will be something that is always changing” (Vignette 3).

#### Learner role

The role of the learner was mentioned most often in response to Vignette 2: Language. Sixty-five percent of respondents mentioned characteristics of the learner that would influence how they would use language. Responses focused on learners’ ability levels and markers such as their English Learner status. A typical comment read, “If a student does not have good language skills they will probably struggle with scientific language” (RS, Vignette 2). Some teachers went further by explaining that it is the teacher’s role to differentiate to meet these different needs. KP wrote, “She needs to be sure that her arrangement of the class and of the assignments is such that her strangest and weakest language students have opportunities to speak and be heard” (KP, Vignette 2). Similarly, when discussing English Learner students, KH mentioned the value of “pictures or drawings for ESL students … to help students to use words successfully while having conversations” (Vignette 2). These trends (i.e., focusing on how to adapt instruction for stronger and weaker students, attending to English Learners) were consistent throughout the responses to Vignette 1: General and Vignette 4: Dialogue, however, when responding to Vignette 3: Negotiation, teachers also mentioned development/ grade level. Although the topic of grade level had not appeared in any prior responses, five responses to Vignette 4 mentioned that negotiation was difficult for children at specific grade levels and/or was developmentally challenging for elementary age students. JD wrote, “It’s not a quick and easy process! I teach first grade, so the teacher needs to help guide and get their conversation in the right direction” (Vignette 4).

#### Big ideas

The role of big ideas was mentioned in 72% of responses to Vignette 1 but was mentioned less often in relation to Vignette 2: Language (18%), Vignette 3: Negotiation (16%), and Vignette 4: Dialogue (20%). Across all four vignettes, when respondents mentioned the big ideas of science, it was always to state that the teacher’s role is to ensure whatever is happening in the classroom is connected to curricular standards, assessment, and/or important science content. For example, when describing how she launches units, KD wrote,

I would tell her to think about her big ideas and what she has to cover in her standards. Then, I would write out the big ideas on large chart paper, and hand out sticky notes. I would instruct them to write down any word that I as associated with the big idea. Then we would categorize them and come up with questions. (Vignette 3)

Similarly, TB wrote “I usually start as a whole group and we brainstorm what we know about the big idea” (Vignette 4) and AS wrote, “Begin with the big idea and allow the students to generate their ideas and lead the discussion (Vignette 1). Although teachers attended to the big idea less when responding to Vignettes 2, 3, and 4 than they did to Vignette 1, within responses that fit this category, there was little variation in how big ideas were described and used.

#### Time and planning

Following Finch (1999), we note that the most common topic teachers raised that was not described in the vignette prompts was the importance of using time wisely. Twenty-nine responses to Vignette 1: General, seven responses to Vignette 2: Language, three responses to Vignette 3: Negotiation, and twenty-four responses to Vignette 4: Dialogue mentioned this topic. In sum, 63 responses of the 474 total responses (13.3%) mentioned time. Many teachers wrote about the need to prioritize student learning despite schedule constraints. In response to Vignette 2, ML began her response by writing “TIME. My transition to [this approach] has required me to rethink how class time is best spent. This is a bit contrary to the down to the minute lesson planning I had done in the past.” KS mentioned that time was important for students as well as teachers. She explained that writing and reflecting on ones’ own learning is important, but “It can be really difficult to allow enough time for this during a class where you are trying to fit so much in” (KS, Vignette 2). Specifically, many teachers explained that they focus on maximizing the time they have. ML wrote, “With about 20 students and only 43 minutes of science every day, I love making the most of every minute we have together” (Vignette 3). Other teachers described disregarding the schedule entirely. LM advocated for taking however much time is needed for dialogue by writing, “If he tries to plan things out too much, then he isn’t going to let the students take the lead and become the catalysts behind the learning … take the time” (Vignette 4). Overall, time was mentioned as a factor that teachers needed to attend to in their teaching and planning.

#### Technology

Although COVID-19 was a shaping factor during this time period, teachers rarely described the role of technology and/or the use of technology as a tool for adaptive teaching when responding to the prompts. Six responses of the 474 total responses (1.3%) mentioned technology. Just one response to Vignette 1 mentioned that text could be shared on a whiteboard or through other technology, but what mattered either way was that “all students can see and participate with as he adds student input/ideas” (SG). Two responses to Vignette 2 mentioned technology in relation to formats of writing. AT puzzled, “Naomi could think about how she wants students to record their thinking in writing. Will she use a journal or have students share writing online...?” (Vignette 2). One response to Vignette 3 and two responses to Vignette 4 similarly mentioned that the format in which students worked would influence what technology they could use. Overall, this topic was not regularly mentioned.

### Vignette analysis results: quantitative

**Fig. 2:**
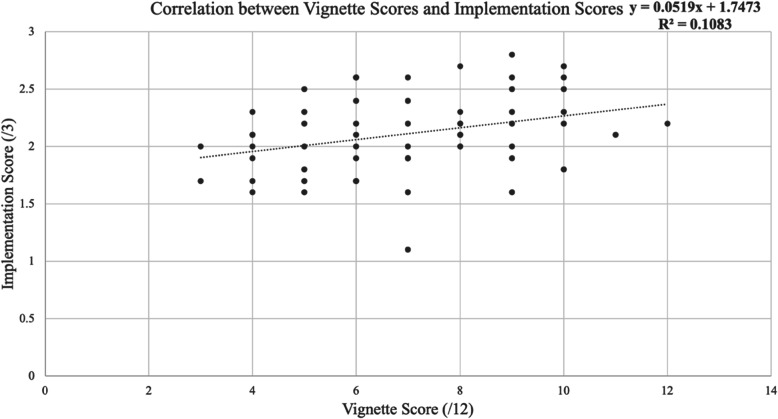
Correlation Between Implementation Scores and Adaptiveness Vignette Scores (*n* = 58)

The ANOVA of teachers’ implementation scores showed significant positive correlation with teachers’ scores on the adaptiveness vignette (Pearson Coefficient = .33 at significance of *P* = 0.01), suggesting a positive relationship between teacher adaptiveness and knowledge generation teaching approaches (Fig. 2; Table 4).

**Table 4:** ANOVA: Regression Statistics comparing Implementation scores and Vignette scores (*n* = 58)

*Regression Statistics*					
Multiple R			0.33		
R Square			0.10		
Adjusted R Square			0.09		
Standard Error			2.06		
	*df*	*SS*	*MS*	*F*	*Significance F*
Regression	1	29.03	29.03	6.80	0.01
	*Coefficients*	*Standard Error*	*t Stat*	*P-value*	
Implementation Score x Vignettes	2.08	0.80	2.60	0.01	

Although the relationship is significant (Pearson Coefficient = .33), the strength of the relationship is not high, suggesting that other contextual factors, including those extending from COVID-19, may have influenced teachers’ ability to enact adaptive practices even if they were capable of describing this approach in a written scenario. To understand these responses further, we turn to the case study results.

### Case study results

LR was a fourth-grade teacher with 12 years of K-12 teaching experience at the start of the 2019–2020 school year. She was a longtime resident of the rural town in a Midwestern state in the U.S. which she taught, which is home to less than 4000 people and is predominantly agricultural. LR’s two children attended school in the same district in which she taught.

### Case study results: adaptiveness

Analysis revealed that LR enacted adaptiveness in two major ways: at the meta-level, by restructuring her course commitments, on the micro-level, through adaptive planning.

#### Macro-level adaptiveness

At the start of the 2019–2020 school year, before the pandemic, LR planned for and taught all subjects. During the 2020–2021 school year, her grade-level teammates decided to pick one content area each to plan for, and LR chose science. In describing the decision to divide by content areas, LR wrote, “It’s been overwhelming to prepare for in-person teaching, hybrid students, and online students. Our team split the work” (Email correspondence; September 16, 2020). LR made a large adaptation to her practice by departmentalizing the content planning.

#### Micro-level adaptiveness

In LR’s lesson plans, and in observations of her teaching, she included smaller regular examples of adaptiveness. Her plans often included phrases such as, “As always, you can adjust the lessons for your brick-and-mortar students based on what works best for you” (Email correspondence; December 16, 2020). This tendency toward adaptive expertise (Hatano & Inagaki, 1986) and option-building was reflected in the decreased length of LR’s lesson planning. When planning in spring 2020, when her classes were fully in-person, LR’s lesson plans averaged 186.5 words per day. Her plans were highly detailed and included lists of material resources and scripts of what she would say to students. For example, her plan for March 10, 2020, explained, “After watching the video, I will ask, ‘What questions do you want to ask about how squid are structured for survival?” During the pandemic, LR’s plans dropped to an average of 47 words, and her plans only included three instances of scripting questions across the 23 lesson plans she submitted from the 2020–2021 school year. Instead, she tended to write statements for herself and her colleagues, such as, “I intentionally left the investigations open so that kids can make their own decisions on how to do it. If you don’t think that’s structured enough, again, feel free to do it your way” (Email correspondence; January 6, 2021). As this example shows, LR often encouraged her colleagues to follow the students’ lead, or to come up with their own plans, both of which require adaptiveness. When asked about the shift to shorter lesson plans, she explained that it was because when teaching during a pandemic, “It’s all in the moment” (Interview, winter 2021). Consistent with theories of adaptiveness (Hatano & Inagaki, 1986; Parsons et al., 2018), she described preparing technology to be ready for whatever happened, rather than having a set plan to follow.

### Case study results: knowledge generation approaches

**Fig. 3:**
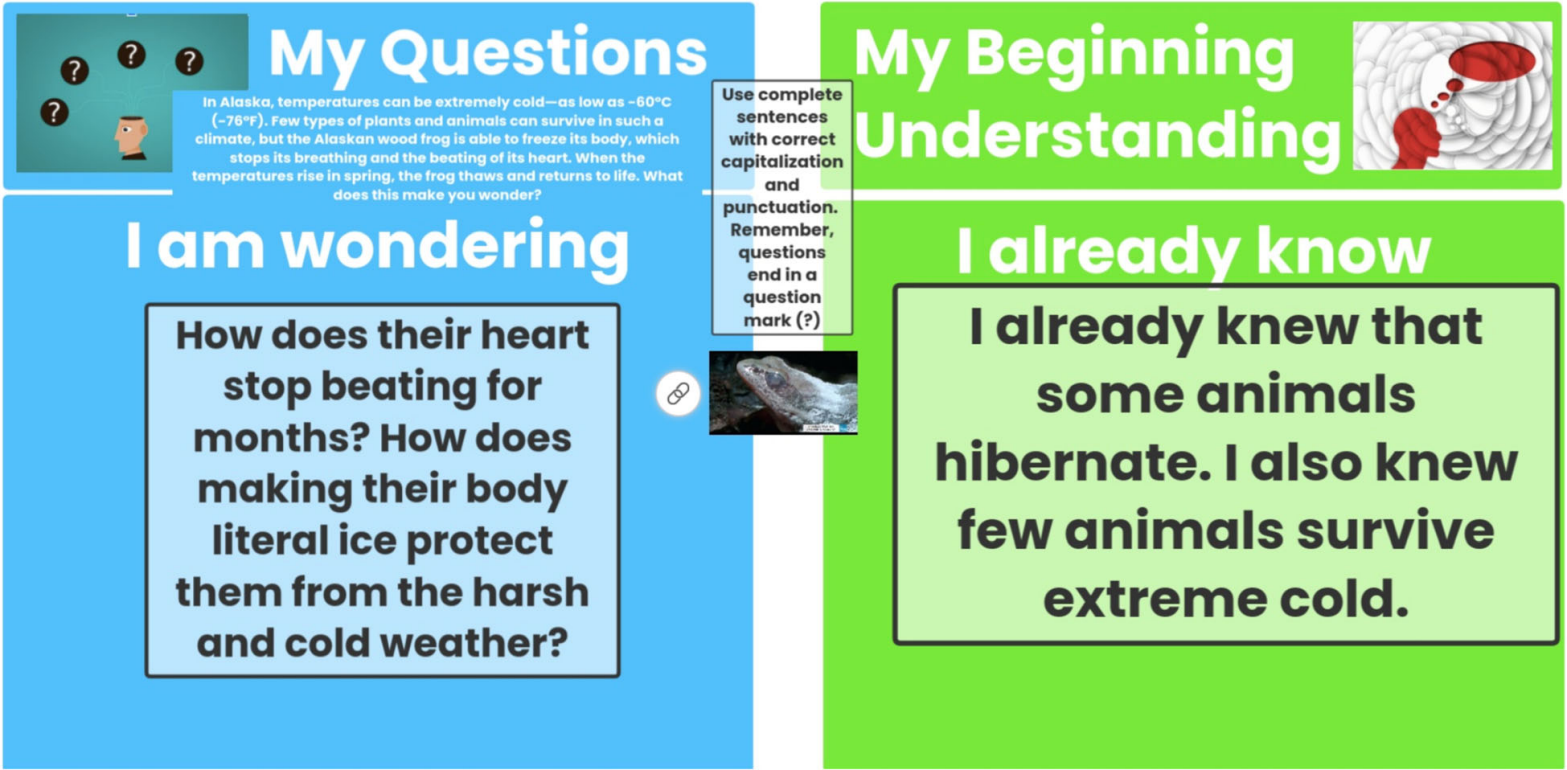
A Screenshot of Students’ Questions from LR’s Class Blog

Analysis of LR’s teaching observations and class blog revealed inconsistent evidence that her planning and teaching process was based on a knowledge generation approach. For example, she often posted content encouraging her students to describe what they already knew and any questions they had, but the planning of investigations based on these questions rarely was noted in observations and on her class blog, particularly at the beginning of the 2020–2021 school year (Fig. 3).

This example shows one student writing that they “already knew that some animals hibernate” but wondered about some species, “how does their heart stop beating for months?” We could not find evidence that this question, while researchable, was pursued. In interviews and emails, LR explained that this was due largely to a lack of material access to resources that were distributed unevenly among students, as well as due to technological constraints that prevented student from communicating their investigation designs with one another. In October, she reached out to the professional development consultant and wrote, “I want to try manipulating sticky notes and working together as a whole group. I can see how the technology can be a barrier, so I’ll keep trying different things” (Email correspondence; October 27, 2020). During her mid-year interview, LR expanded on what had made a knowledge generation approach difficult at the start:

You know, creating things. … We were doing that a little bit in the beginning, but then we also didn’t have all of our learners here. We felt like we had to modify some things so that they were having the same experience at home as we were having here. So, we felt like we had to, you know, tamp down some of our things to make it fair because that was what our district was offering. [The district asked] Was it going to be the same at home as it is at school? We know it’s not.

As LR explained, she had students ask questions and pose ideas for how they might investigate their questions regardless of teaching format. However, actually conducting investigations required materials that her remote learners could not access, creating a barrier for her as a teacher who valued educational equity.

When LR was finally able to be observed teaching in person in May 2021, she had returned to supporting the student-led investigations reflective of a knowledge generation approach. This class, which involved flower dissection, began with students generating questions and LR asking, “I want us to look at our questions. What are some of these questions that we can answer today?” (Field notes; May 18, 2021). As the students excitedly moved around the room – dissecting, measuring, and recording data to answer their questions – one realized that he had confused petals and leaves when writing in his science notebook. “It’s OK to revise. That’s how we learn,” LR was overheard saying (Field notes; May 18, 2021), reflecting her adaptive stance toward all things.

## Discussion

Although it was evident prior to the pandemic that adaptiveness is key to effective science teaching (Collie et al., 2020), COVID-19 highlighted the complex ways adaptiveness functions moment-to-moment (Hatano & Inagaki, 1986). This study explored how teacher adaptiveness relates to elementary science teachers’ use of knowledge generation approaches in teaching altered by the COVID-19 pandemic. It revealed that science teacher adaptiveness enables teachers to enact NGSS-aligned approaches regardless of the format in which they teach. In this study, effective planning involved constructing options and noting areas requiring adaptiveness, and effective teaching involved ignoring the clock. In particular, teachers reported the need to let go of rigid schedule demands to focus on student learning, and they recognized that learning to teach adaptively through the use of knowledge generation approaches is a long, difficult process.

This study also demonstrated that the realities of COVID-19 altered the contexts for student investigations in science teaching. In particular, the case study revealed that teachers’ willingness to rely on student-designed investigations depends in part on student access physical resources needed to conduct such investigations. Here, it was clear that this access deteriorated during remote and virtual learning but returned during in-person teaching. Although teachers rarely mentioned technology in their responses to the vignettes, the case study showed that technology was a major factor influencing teachers and students’ lived realities. Teachers’ lack of attention to technology in their vignette responses may have foreshadowed challenges in their classrooms, since research suggests they were called upon to use technology in new ways during this timeframe (Svrcek et al., 2021). Thus, the current study suggests the importance of the previously unexplored interplay between adaptiveness, knowledge generation approaches, instructional planning, and physical/technological resources.

### Limitations

In interpreting the value of this study, we note that the context of interest (i.e., the pandemic) is also a source of this work’s methodological limitations. Had COVID-19 not disrupted schooling in spring 2020, we might have been able to conduct observations of all 119 teachers in the larger study. Furthermore, the difficulty of teaching during the 2020–2021 school year limited our data collection options, and it is possible that our results might have differed had different teachers been involved at each step (e.g., the professional development as a whole, the observations of implementation, and the case study). In particular, including additional teachers in the case study component of this research would have improved the transferability of the findings.

As it stands, this article represents the outcome of methodological decisions made to gather data with minimal intrusiveness and maximum beneficence (Creswell, 2013). By using a single case design in addition to the vignettes and classroom observations, we ensured that all 119 teachers’ participation in the study did not detract from their attention to their families, students, and their own human needs. We argue that research participants’ well-being should be prioritized over the quest for knowledge at all times, but especially during a pandemic.

### Implications

This research was designed to answer the question: In teaching altered by the COVID-19 pandemic, how does elementary science teacher adaptiveness relate to the use of knowledge generation approaches? First, this research suggests that unexpected shifts in learning formats can create situations in which teachers prepare to be flexible, rather than script rigid lessons and deviating from them only where needed. This indicates that more professional learning is needed to promote the type of planning consistent with adaptiveness. Previous studies have noted that more experienced teachers tend to engage in more sophisticated cognition when planning for instruction than less experienced teachers (Contreras et al., 2020). It is possible that the process of planning for adaptive teaching in ways that use language, negotiation, and dialogue, requires deeper thinking as teachers anticipate what might unfold. The fact that LR’s lesson plans grew shorter during this time period does not necessarily mean she thought less about how to prepare for instruction. In addition, particularly in their vignette responses, teachers mentioned that effective science lessons do not always take the same amount of time from day to day, and flexible scheduling is necessary to enact knowledge generation approaches. The relationship between teacher cognition, planning, and adaptive teaching is an important area of future study.

The relationship between the use of language tools and adaptiveness also bears unpacking. In responding to vignettes, many teachers described the role of big ideas, anchoring phenomena, and/or science content when replying to a generic prompt, but lost sight of this element when describing how they would use a particular tool. This may suggest the complexity of adaptive teaching, which requires the ability to juggle multiple instructional elements. In addition, teachers’ consideration of developmental needs/grade level in relation to student negotiation, but not language or dialogue, may reflect the ways teachers’ views of learning influence the way they teach. Although the amount of data we collected on this topic is small, further research into ways teachers conceptualize negotiation across childhood developmental trajectories, and research on the professional development can support teachers’ ability to attend to multiple instructional elements, is merited. In addition, as schools recover from the pandemic, it is important for administrators and policymakers to support this type of NGSS-aligned teaching. It is possible that fears of COVID-19-related learning loss may drive a policy agenda aimed at increased accountability and skills remediation, which runs counter to the type of NGSS-aligned (2013) and OECD-promoted (2018) teaching seen in this study.

Finally, we note that in attempting to elevate the importance of adaptiveness, we are not denying the importance of content knowledge, pedagogical ability, or knowledge of instructional technology. In fact, we theorize that a strong relationship exists between the development of all three, and that what may drive teachers’ actions is how they understand the combination of their abilities. This study’s results pushed us to wonder how professional development that combines these areas might take form. For example, what could teachers learn through observing teacher educators taking an adaptive approach to providing online, technology-facilitated professional development? Further research is needed to theorize the additional roles adaptiveness could have in professional development.

## Conclusions

Engelbrecht et al. (2020) asked whether 2020 will be remembered as the year in which education was changed. Our answer is yes – and no. The world changed dramatically, but our core goal of promoting scientific literacy by improving science teaching has remained, and perhaps became even more important, during this time. As a result of the pandemic, people worldwide were called on to make daily decisions (e.g., mask or no mask, stay home or go out) that impacted public health. Now, more than ever, the value of every person knowing how knowledge is generated in science is apparent. While we cannot erase the negative impacts of COVID-19, we can put our hope for the future in improvements in science education, including those suggested by this study. This research adds to the growing body of evidence that suggests that teacher adaptiveness is key in the use of knowledge generation approaches for learning, and that myriad contextual factors are at play as teachers navigate this challenging work both during the pandemic and beyond.

## Supplementary Information

**Additional file 1: Supplementary Table 1.** Teacher Observation Implementation Guide. **Supplementary Table 2.** Vignette Tasks**. Supplementary Table 3.** Content Analysis Rubric for Vignette Tasks.

## Data Availability

The datasets generated and/or analyzed during the current study are not publicly available due to institutional restrictions, but are available from the corresponding author on reasonable request.

## References

[CR1] Allen, M. H., Matthews, C. E., & Parsons, S. A. (2013). A second-grade teacher’s adaptive teaching during an integrated science-literacy unit. *Teaching and Teacher Education*, *35*, 114–125.

[CR2] Bingimlas, K. A. (2009). Barriers to the successful integration of ICT in teaching and learning environments: A review of literature. *Eurasia Journal of Mathematics, Science and Technology Education*, *5*, 235–245. 10.12973/ejmste/75275

[CR3] Boon, M., & Van Baalen, S. (2019). Epistemology for interdisciplinary research: Shifting philosophical paradigms of science. *European Journal for Philosophy of Science*, *9*, 16. 30873248 10.1007/s13194-018-0242-4 PMC6383598

[CR4] Briggs, C. L. (2010). What were they THINKING? Nursing students’ thought processes underlying pain management decisions. *Nursing Education Perspectives*, *31*(2), 84–88. 20455363

[CR5] Collie, R. J., Granziera, H., Martin, A. J., Burns, E. C., & Holliman, A. J. (2020). Adaptability among science teachers in schools: A multi-nation examination of its role in school outcomes. *Teaching and Teacher Education*, *95*, 1–11.

[CR6] Contreras, K., Arredondo, C., Díaz, C., Inostroza, M. J., & Strickland, B. (2020). Examining differences between pre- and in-service teachers’ cognition when lesson planning. *System*, *91*, 1–14. 10.1016/j.system.2020.102240.

[CR7] Creswell, J. W. (2013). *Qualitative inquiry & research design*. SAGE Publications.

[CR8] Engelbrecht, J., Borba, M. C., Llinares, S., & Kaiser, G. (2020). Will 2020 be remembered as the year in which education was changed? *ZDM*, *52*(5), 821–824. 10.1007/s11858-020-01185-3. 32837580 10.1007/s11858-020-01185-3 PMC7374655

[CR9] Finch, J. (1999). The vignette technique in survey research. In C. Barter, & E. Renold (Eds.), *The use of vignettes in qualitative research*, (pp. 105–114).

[CR10] Gewin, V. (2020). Five tips for moving teaching online as COVID-19 takes hold. *Nature*, *580*(7802), 295–296. 10.1038/d41586-020-00896-7. 32210377 10.1038/d41586-020-00896-7

[CR11] Hand, B., Chen, YC. & Suh, J.K. (2021). Does a Knowledge Generation Approach to Learning Benefit Students? A Systematic Review of Research on the Science Writing Heuristic Approach. *Educational Psychology Review* 33, 535–577.10.1007/s10648-020-09550-0

[CR12] Hatano, G., & Inagaki, K. (1986). Two courses of expertise. In Child Development and Education in Japan (pp. 263-272) H. W. Stevenson, H. Azuma, and K. Hakuta, Eds., W.H. Freeman & Co.

[CR13] Hodges, C., Moore, S., Lockee, B., Trust, T., & Bond, A. (2020). The difference between emergency remote teaching and online leaning. Educause review*.* Retrieved from: https://er.educause.edu/articles/2020/3/the-difference-between-emergency-remote-teaching-and-online-learning

[CR14] Keys, C. W., Hand, B., Prain, V., & Collins, S. (1999). Using the science writing heuristic as a tool for learning from laboratory investigations in secondary science. *Journal of Research in Science Teaching*, 36(10), 1065–1084. 10.1002/(SICI)1098-2736(199912)36:10<1065::AID-TEA2>3.0.CO;2-I

[CR15] Loughland, T., & Alonzo, D. (2018). Teacher adaptive practices: Examining links with teacher self-efficacy, perceived autonomy support and teachers’ sense of adaptability. *Educational Practice and Theory*, *40*(2), 55–70.

[CR16] Martin, F., Sun, T., & Westine, C. D. (2020). A systematic review of research on online teaching and learning from 2009 to 2018. *Computers & Education*, *159*, 104–109. 10.1016/j.compedu.2020.104009 PMC7480742 32921895

[CR17] McNeill, K. L., Gonzalez-Howard, M., Katsh-Singer, R., & Loper, S. (2016). Pedagogical content knowledge of argumentation: Using classroom contexts to assess high-quality PCK rather than pseudoargumentation. *Journal of Research in Science Teaching*, *53*, 261–290.

[CR18] Mertens, D. M. (2015). *Research and evaluation in education and psychology: Integrating diversity with quantitative, qualitative, and mixed methods*, (4th ed., ). SAGE.

[CR19] Mitchell, C. J. (1984). Typicality and the case study. In R. F. Ellens (Ed.), *Ethnographic research: A guide to general conduct (pp. 238–241)*. Academic.

[CR20] NGSS Lead States (2013). *Next generation science standards: For States, by States*. The National Academies Press.

[CR21] Organization for Economic Cooperation and Development (OECD). (2018). Preparing our youth for an inclusive and sustainable world: The OECD PISA global competence framework. https://www.oecd.org/education/Global-competency-for-an-inclusive-world.pdf.

[CR22] Parsons, S. A., Vaughn, M., Scales, R. Q., Gallagher, M. A., Parsons, A. W., Davis, S. G., … Allen, M. (2018). Teachers’ instructional adaptations: A research synthesis. *Review of Educational Research*, *88*(2), 205–242.

[CR23] Saldaña, J. (2013). *The coding manual for qualitative researchers*, (2nd ed., ). SAGE.

[CR24] Strauss, A., & Corbin, J. (1998). *Basics of qualitative research: Grounded theory procedures and techniques*. Sage.

[CR25] Svrcek, N. S., Rath, L., Olmstead, K., & Colantonio- Yurko, K. (2021). “We are still putting out fires”: Considering educator intentionality in remote instruction during the COVID-19 pandemic. *Education and Information Technologies*, *27*, 407–428. 34483705 10.1007/s10639-021-10679-w PMC8406388

[CR26] Tang, K.-S. (2020). The use of epistemic tools to facilitate epistemic cognition & metacognition in developing scientific explanation. *Cognition and Instruction*, *38*(4), 474–502.

[CR27] van Broekhuizen, L. (2016). The paradox of classroom technology: Despite proliferation and access, students not using technology for learning. AdvancED.

[CR28] Vaughn, M., & Parsons, S. A. (2013). Adaptive teachers as innovators: Instructional adaptations opening spaces for enhanced literacy learning. *Language Arts*, *91*(2), 81–93.

[CR29] Vogt, F., & Rogalla, M. (2009). Developing adaptive teaching competency through coaching. *Teaching and Teacher Education*, *25*, 1051–1060.

[CR30] Yin, R. K. (2013). *Case study research: Design and methods*, (5th ed., ). Sage Publications.

