# Supplementary material for: “It’s all in the moment”: a mixed-methods study of elementary science teacher adaptiveness following professional development on knowledge generation approaches
Source: Discip Interdscip Sci Educ Res. 2022 Apr 5;4(1):12. doi: 10.1186/s43031-022-00052-3 (PMC8980771; doi:10.1186/s43031-022-00052-3)
Supplement: Supplementary file 1 — Additional file 1: Supplementary Table 1. Teacher Observation Implementation Guide. Supplementary Table 2. Vignette Tasks. Supplementary Table 3. Content Analysis Rubric for Vignette Tasks. [file 43031_2022_52_MOESM1_ESM.docx]

**Supplementary Table 1**

*Teacher Observation Implementation Guide*

| **Teacher Practices** | | **NA** | **Low**   | **Medium**   | **High**   |
| --- | --- | --- | --- | --- | --- |
| **1** | **Student Voice**  Creating an environment that gives students a voice to engage in knowledge generation. | No Evidence;  Not Observed | The teacher dominates classroom talk;  There are some initiations to T-S dialogue | T-S dialogue is very common;  Students’ ideas/questions are reflected back to students;  S-S dialogue is rare;  Students are encouraged to be engaged actively in negotiation | S-S dialogue is very common;  T-S dialogue is only for guidance;  Students are given authority to construct/generate knowledge;  Students are engaged actively in negotiation |
| **2** | **Teacher Questioning**  Utilizing questioning to promote students’ deeper cognition | No Evidence;  Not Observed | Feedback for questions and responses as “right” or “wrong”;  Relatively few open-ended questions;  I-R-E is a common pattern   | The teacher asks mostly open-ended questions;  The teacher encourages questions from students;  The I-R-F pattern is common | Students ask more questions and challenge others, need not wait for teacher to pose questions;  Questions focus on provoking students’ interest or expanding the direction of discussions;  The I-R-F pattern is common |
| **3** | **Big Ideas**  Determining/presenting big ideas and diagnosing/unpacking prior knowledge via dialogical interactions | No Evidence;  Not Observed | Big ideas are presented without any discussion;  Ideas are written on the board/are made visible to all students;  Few questions are asked to unpack prior knowledge | Some discussions on big ideas are conducted with students, but the teacher presents/determines the big ideas;  Students are encouraged to ask questions and initiate dialogue to unpack prior knowledge | Strong student discussions on big ideas are conducted (with the teacher acting only as moderator);  Students are allowed to restate big idea through their expression (authorship/ownership);  The students and teacher work together to unpack prior knowledge;  Students are encouraged to make explicit connections to big ideas |
| **4** | **Language Use**  Promoting the flexible use of language for knowledge generation | No Evidence;  Not Observed | The teacher and textbook guide language use;  The teacher uses limited multimodal representation   | Everyday language is allowed;  Students use MMR moderately, but the teacher still often leads it   | Everyday language is common;  Scientific vocabulary is introduced when needed;  Smooth transitions are made between everyday and scientific language;  Students regularly use multimodal representation; the teacher encourages students to connect modes and link ideas   |
| **5** | **Writing to Learn**  Using writing as a learning tool through varied approaches | No Evidence;  Not Observed | The teacher instructs students to follow the SWH framework like a simple template;  Students follow templates for writing without further data on purpose or audience | Students use the SWH framework flexibly, rather than formulaically;  Students recognize or state the purpose and intended audience for their writing | Students use the SWH framework flexibly, rather than formulaically, with an emphasis on learning from one’s writing;  Students discuss their choices of audience and purpose when they prepare to write;  The teacher encourages students to reflect on prior writing and recognize how ideas change |
| **6** | **Negotiation Structures**  Promoting the use of question- design-claim-evidence (Q-D-C-E) cohesively as a structure for a scientific argument |   No Evidence;  Not Observed | There is little apparent use of Q-D-C-E argument structures as a template for replicating steps;  The teacher prompts the use of argumentation structure | There is moderate apparent use of Q-D-C-E;  The teacher and students develop argumentation components together;  Q-D-C-E linkage is strengthened | Students prompt the use of argument structures when discussing investigations;  Students develop their own designs and arguments based on their questions;  Claims and evidence are coherent with questions and the result of their design |
| **7** | **Student Negotiation**  Encouraging students to adopt, critique, and defend ideas to generate each component of a scientific argument | No Evidence;  Not Observed | The teacher challenges students to elaborate their thoughts on argument components;  Students’ initiations are dependent on teacher feedback;  The teacher dominates argumentation in the classroom | Students initiate critiques and defend their claims on science argument components;  The teacher provides some direction to guide students;  Students start to elaborate Q-D-C-E independent of the teacher | Students critique or defend each Q-D-C-E part;  Students help each other generate argument components;  The teacher participates only in argument development;  Persuasion and challenges are the main characteristics |
| **8** | **Group structure**  Promoting students’ engagement in dialogic interactions as small group and whole class during work/talk/discussion | No Evidence;  Not Observed | Some group discussions are conducted around what the teacher says—not very productive;  Whole class discussions are held with some students’ participation | Group work dominates the class, with students working collaboratively;  Whole class discussions are conducted with high participation, including teacher participation;  Students are encouraged to contribute in group endeavors | Every student contributes to conversations;  Small-group and whole-class discussions are very distinct;  The teacher participates through shadowing, but students fully direct discussions without teacher contributions |
| HIGH 🡨-------------------------------------------Teacher Visibility ------------------------------------------------------🡪 LOW | | | | | |
| LOW 🡨------------------------------------------- Student Visibility ------------------------------------------------------🡪 HIGH | | | | | |

**Supplementary Table 2**

*Vignette Tasks*

| **Administration Instructions** | Participants receive one vignette prompt per sitting through a Google Form. Participants are instructed as follows: “Please respond to the vignettes in writing. You have 10 minutes to respond.” |
| --- | --- |
| **Vignette 1: General** | Thomas has just finished his first Science Writing Heuristic workshop and is uncertain how to prepare for teaching science. He knows he won’t be able to anticipate all the different ideas his students will share or where the investigations may go. Please describe to Thomas how he could think about science teaching as he prepares. Include any steps he should take, and make your thinking transparent so he knows why these steps are important. |
| **Vignette 2: Language** | Naomi completed a Science Writing Heuristic workshop last summer. She thinks her science teaching went OK this year, but she wants to make better use of language. Please describe to Naomi how she could think about language as she prepares. Include any steps she should take, and make your thinking transparent so she knows why these steps are important. |
| **Vignette 3: Negotiation** | Emma completed a Science Writing Heuristic workshop last summer. She thinks her science teaching went OK this year, but she wants to make better use of negotiation. Please describe to Emma how she could think about negotiation as she prepares. Include any steps she should take, and make your thinking transparent so she knows why these steps are important. |
| **Vignette 4: Dialogue** | Kevin has just finished his first Science Writing Heuristic workshop and is uncertain how to prepare for teaching science. He is particularly uncertain about dialogic interaction. Please describe to Kevin how he could think about dialogic interaction as he prepares. Include any steps he should take, and make your thinking transparent so he knows why these steps are important. |

**Supplementary Table 3**

*Content Analysis Rubric for Vignette Tasks*

| **Category** | **Descriptors for Current Vignette Analysis** | **Related Category from Previous Vignette** | **Example from Current Data Set** | **Score** |
| --- | --- | --- | --- | --- |
| **Teacher** | These responses address teachers’ need for “flexibility,” “fluidity,” or “adaptiveness” and normalize the challenges of knowledge generation approaches. Responses may include attention to specific language tools (writing, talking, drawing, diagramming), dialogue (discussion, questioning), and negotiation, argument, or questions, claims, evidence. | “Depth of reflection” and “Practical realisation of highly differentiated quality” (Vogt & Rogalla, 2009, p. 1055) | “I think maybe one of the hardest parts is letting go of a perfectly planned lesson, and giving in to what students are able to do with the materials the kids have access to” (A.P., Vignette 1). | _/1 |
| **Learner** | These responses address what it means to “learn” and what “learning is,” or describe students’ control over their “learning” process. They also may use words like “know” or “knowledge” when discussing the role of students’ existing ideas. These responses may address “control” and “freedom” in relation to learning. | “Orientation towards individually diverse learning processes of each student” (Vogt & Rogalla, 2009, p. 1055) | “Let the students lead the direction of the learning. Let them ask questions and answer them with a question to give the power of learning to the kids.” (A.D., Vignette 1) | _/1 |
| **Big Ideas** | These responses address fixed ideas and concepts. They may describe “big ideas,” “curricular standards,” “content,” or guiding, driving, or anchoring “phenomena.” | “Orientation towards learning for understanding” (Vogt & Rogalla, 2009, p. 1055) | “Look at the standards he plans to teach and what the bundle looks like. He could start with the big idea or present a phenomenon to get his students thinking” (AT, Vignette 3). | _/1 |
